# Survival Status and Predictors of Mortality among Newborns Admitted with Neonatal Sepsis at Public Hospitals in Ethiopia

**DOI:** 10.1155/2020/8327028

**Published:** 2020-09-19

**Authors:** Samuel Dessu, Aklilu Habte, Tamirat Melis, Mesfin Gebremedhin

**Affiliations:** ^1^Department of Public Health, College of Medicine and Health Sciences, Wolkite University, Wolkite, Ethiopia; ^2^Department of Public Health, College of Medicine and Health Sciences, Wachamo University, Ethiopia; ^3^Department of Public Health College of Medicine and Health Sciences, Arba Minch University, Ethiopia

## Abstract

**Background:**

One-fourth of neonatal death is due to neonatal sepsis and nearly 98% of these deaths are occurring at low- and middle-income countries. In Ethiopia, forty percent of under-five mortality occurs during the neonatal period, of which neonatal sepsis accounts for 30-35% of neonatal deaths next to prematurity and its complications. On the other side, among the survived neonates with neonatal sepsis, there exist as vulnerable to short and long-term neurological and developmental morbidity impacting the overall productivity of the child as adult.

**Methods:**

A longitudinal prospective cohort study was conducted among selected 289 neonates with neonatal sepsis who were admitted in the neonatal intensive care unit at public hospitals in Ethiopia from 1^st^ March 2018 to 31^st^ December 2019. Data were entered into Epi data version 3.02 and exported to SPSS V 25 for analysis. The Kaplan-Meier survival curve together with log-rank test was used to estimate the survival time of the neonates. Variables which had *p* value < 0.05 in multivariable analysis using the cox proportional hazard model were declared as statistically significant predictors of mortality.

**Results:**

The study was conducted with a total of 289 neonates admitted with neonatal sepsis. The cumulative proportion of surviving at the end of the fourth day was 99.5%, and it was 98.2% at the end of the fifth day. In addition, it was 96.6%, 93.5%, and 91.1% at the end of the sixth, seventh, and eighth day, respectively. The incidence of mortality was 8.65 per 100 neonates admitted with neonatal sepsis. Having a history of intrapartum fever (AHR: 14.5; 95% CI: 4.25, 49.5), history of chorioamnionitis (AHR: 5.7; 95% CI: 2.29, 13.98), induced labor (AHR: 7; 95% CI: 2.32, 21.08), and not initiating exclusive breastfeeding within one hour (AHR: 3.4; 95% CI: 1.34, 12.63) were the independent predictors of mortality.

**Conclusion:**

The survival status of neonates among neonates admitted with neonatal sepsis was high at the early admission days and high cumulative proportion of death as the admission period increased. The risk of mortality was high among the neonates with early onset of neonatal sepsis as compared with late onset of neonatal sepsis and history of intrapartum fever, history of diagnosed chorioamnionitis, onset of labor, and EBF initiation within one hour were the independent predictors of mortality among neonates admitted with neonatal sepsis.

## 1. Background

Neonatal sepsis is a condition defined as a clinical syndrome in the presence of or as a result of suspected or proven infection in neonates. It presents with clinical features of temperature instability, respiratory problems, feeding intolerance, and isolation of bacteria or other pathogens from the bloodstream [1, 2]. It is classified as early-onset neonatal sepsis (occurs before three to seven days) and late-onset neonatal sepsis (occurs after seven days) [3, 4].

One fourths of neonatal deaths are due to neonatal sepsis, and nearly 98% of these deaths are occurring at low- and middle-income countries [5–7]. Its incidence varies from health institution to health institution and within the same health institution at varied times and depends on factors predisposing to infection [8]. It is one of the major causes of morbidity and mortality in neonates worldwide, in spite of recent advances in the healthcare system and is an ongoing major global public health challenge [9–11]. Neonatal sepsis causes a vigorous public health challenge for the Sub-Saharan Africa with a significant related economic crisis [12].

The proportion of bloodstream infection (BSI) in newborns was three to twenty times higher in developing countries, which is approximately 50% of the neonates in hospital (NICUs) developed neonatal infection [13]. The case fatality rates of neonatal sepsis may reach 52% contributing for almost one million deaths and are responsible for about 30-50% of the total neonatal deaths. In addition, sepsis can be prevented through timely recognition, rational antimicrobial therapy, and aggressive supportive cares [14, 15].

In Ethiopia, forty percent of under-five mortality was occurring during the neonatal period, of which neonatal sepsis accounts for 30-35% of neonatal deaths next to prematurity and its complications [16]. On the other side, among the survived neonates with neonatal sepsis, there exist as vulnerable to short- and long-term neurological and developmental morbidity impacting the overall productivity of the child as an adult [13]. Due to the varied nature of the microorganism causing neonatal sepsis varies from place to place and changes over time even in the same place which could be attributed to the varied pattern of antimicrobial provision and changes in lifestyle [17, 18].

The most common cause of mortality among neonates admitted with neonatal sepsis in Sub-Saharan Africa has been identified as sociodemographic factors, maternal, obstetrical, and neonatal factors such as maternal age, history of chorioamnionitis, APGAR score, and exclusive breastfeeding (EBF) initiation [1, 4, 8, 9]. Therefore, this study was conducted to determine the survival time and predictors of mortality among neonates admitted with neonatal sepsis at public hospitals in Ethiopia.

## 2. Methods

### 2.1. Study Design, Area, and Period

A longitudinal prospective cohort study was conducted at Arba Minch General hospital, Sawla General Hospital, and Chencha district hospital from 1^st^ March 2018 to 31^th^ December 2019, to assess the survival time, incidence, and predictors of mortality among neonates admitted with neonatal sepsis. The study was conducted with all the neonates admitted with neonatal sepsis within the time frame. All neonates admitted to the neonatal intensive care unit (NICU) with diagnosed neonatal sepsis and who had age less than 28 days were the source populations.

The follow-up period was initiated at admission to the NICU on 1^st^ March 2018 and closed on 31^st^ December 2019. The neonates were followed until they had a maximum age of 28 days. The follow-up was completed if the study subject was either died, recovered, loss to follow-up, transferred to another institution, and follow-up time was completed without outcome happening. In addition, any neonate, who withdraw a treatment, discharged alive, transferred out, and did not yet developed the outcome at the end of the follow-up period, was termed as censored. The study was an open cohort study, and any newborn with neonatal sepsis within the study period was entered and leaves the study. Within this time frame, there were a total of 289 neonates with diagnosed neonatal sepsis, and the study was conducted among all the diagnosed cases.

### 2.2. Variables

The dependent variable was time to death, and the independent variables were sociodemographic factors (age of the newborn, sex of the newborn, maternal age, marital status, religious status, educational status of the mother, maternal occupational status, family size, and estimated monthly income of the family), obstetrics factors (number of ANC visits, gravidity, parity, place of delivery, delivery attendant, number of per vaginal digital examinations, history of foul-smelling liquor, history of pregnancy-induced hypertension, history of bleeding during pregnancy, history of UTI/STI, history of meconium-stained amniotic fluid, history of intrapartum fever, history of diagnosed chorioamnionitis, time of rupture of membrane, onset of labor, mode of delivery, and gestational age), and neonatal factors (first minute APGAR score, fifth minute APGAR score, cry immediately at birth, resuscitated at birth, kept in KMC within one hour, birth weight, and EBF initiated within one hour).

### 2.3. Definition of Terms

Clinical sepsis: sepsis in which blood culture is not performed, not detected, or for which the physician institutes treatment for sepsis but this study was not focused on clinical sepsis.

Early-onset neonatal sepsis: sepsis diagnosed among neonates at the age of three to seven days.

Late-onset neonatal sepsis: sepsis diagnosed among neonates at the age of seven days or more.

### 2.4. Data Collection Tool and Procedure

Data were collected by trained data collectors using a structured checklist. Both primary and secondary data were used. Data quality was assured by caring out the careful design of data extraction tools and training of both the data collectors and supervisors. Over more, pretesting was ensured in 5% of the populations to improve the skill of data collectors and to ensure the consistency of the data extraction tool. The validity and reliability of the tool was ensured.

In this study, neonatal sepsis was diagnosed as either of the clinical manifestations (changes in temperature, feeding problem, fussiness, lack of energy, high-pitched cry, yellow, blue, or pale skin, bruising or bleeding, cool, clammy skin, skin rashes, fast breathing, problems breathing, or periods of no breathing, vomiting, and diarrhea) and at least one positive laboratory test for a bacterial pathogen (It could be positive bacterial culture result/polymerase chain reaction (PCR)/gram-staining/latex agglutination tests/antigen-antibody detection for bacteria). The hospitals used BacTec (Becton Dickinson Microbiological System, Maryland) for bacterial culture.

### 2.5. Data Processing and Analysis

Epi data version 3.02 was used to enter the data, code the data, edit the data, and clean the data and finally exported to SPSS version 25 to conduct statistical analysis. The empirical relationship among the outcome variable and the explanatory variables was determined using bivariate statistical analysis. Both Crude hazard ratio (CHR) and adjusted hazard ratio (AHR) together with the corresponding 95% confidence interval and *p* value were used to assess the strength of association and statistical significance. The Kaplan-Meier survival curve together with log-rank test was fitted to determine the survival time. Variables which had *p* value < 0.05 in bivariate analysis were considered as candidate for multivariable analysis and variables which had *p* value < 0.05 in multivariable cox regression analysis were considered as statistically significant.

### 2.6. Ethical Consideration

Ethical clearance was obtained from Arba Minch University, college of medicine and health sciences ethical review board. In addition, a permission letter was obtained from the Arba Minch University. A written consent was obtained directly from the mothers. Mothers who did not read and write were informed about the information written on the informed consent. Over more, mothers were informed about the objective and significance of the study prior to the data collection. Appropriate measures were applied to ensure the confidentiality of the data.

## 3. Results

### 3.1. Sociodemographic Characteristics

In this study, a total of 289 neonates with their index mothers were involved. The minimum and maximum age of the neonates was one day and 27 days, respectively, with a mean of 4.66 ± 6.2 days. Nearly four-fifths (80%) of the neonates had age less than 7 days. Among the neonates who died with neonatal sepsis, 15 (60%) of them died within the first seven days (early onset of neonatal sepsis). Regarding the sex of the neonates with neonatal sepsis, 180 (62.3%) were males and among them, 16 were died, which accounted for 64.0% of dead neonates. One hundred and nine (37.70%) of the neonates were females, of which 164 were survived, which accounted for 62.1% of the survived neonates at the end of the follow-up period.

The minimum age of the mother was 18 years old, and the maximum age was 42 years old with a mean of 27.68 ± 5.77 years. Among the total respondents, 38 (13.1%), 201 (69.6%), and 50 (17.3%) of the mothers were categorized under <20 years old, 20-34 years old, and >34 years old, respectively. In considering the mortality, the majority of neonates (44%) died among mothers having age > 34 years old. Nearly, one-tenth (9.3%) of the mothers were never married and the remaining 262 (90.7%) were married. Eighteen (72.8%) of the dead and 9 (3.4%) of the survived were delivered from never-married mothers. The majority of the respondents (42.6%) were Orthodox in their religion while the remaining 54 (18.7%), 95 (32.9%), and 11 (5.9%) were Muslims, protestants, and others, respectively. Regarding the maternal educational status, 39 (13.5%) were unable to read and write while read and write, grade 1-8, grade 9-12, and college and above accounted 66 (22.8%), 94 (32.8%), 59 (20.4%), and 31 (10.7%), respectively. Regarding the maternal occupational status, both housewife and civil servant accounted for nearly one-tenth of the mothers.

The majority of the mothers (45.3%) had family size less than four while 91 (31.5%) of the mothers had 4-6 family size and 67 (23.2%) had family size more than six. In considering the neonatal mortality, 60% of them died among the categories having family size more than six. Regarding the estimated monthly income of the families, 104 (36%) had more than 2600 Ethiopian birr. The remaining 91 (31.5%), 72 (24.9%), and 22 (7.6%) of them had 2000-2599, 1400-1999, and less than 1399 Ethiopian Birr, respectively. Most of the deaths (40%) were observed among the families having monthly income less than 1399 Ethiopian Birr (Table 1).

### 3.2. Obstetrics Related Variables

In considering the number of visits of antenatal care, around one-tenth (10.7%) of the mothers had no antenatal visits. Similarly, 84 (29.1%) of the mothers had one visit during this pregnancy, while nearly one-fourth (24.9%) of the mothers had two visits. The remaining 61 (21.1%) and 41 (14.2%) of the mothers had three and four visits, respectively. In undertaking the dead neonates, 36% of them were from mothers with no antenatal visits, followed by one antenatal visit (24%). The majority of the mothers (46.7%) had more than four instances of pregnancies followed by one or two instances (31.8%).

Regarding the place of delivery, around three fourths (765%) of the newborn were delivered at a health institution and the remaining 68 (23.5%) were delivered at home. Nearly half (49.1%) of the deliveries were attended by health professionals, while 79 (27.3%), 37 (12.8%), and 31 (10.7%) of deliveries were attended by health extension workers, trained traditional birth attendants, and relatives, respectively. In undertaking per vaginal digital examination, for 171 (59.2%) of the mothers, four and above number of per vaginal examinations were performed. Among the mothers, 45 (15.6%) of them had a history of foul-smelling liquor.

In considering health problems during pregnancy, 71 (24.6%), 75 (25.9%), 54 (18.7%), 57 (19.7%), and 34 (11.8%) of the mothers had a history of pregnancy-induced hypertension, history of bleeding during pregnancy, history of urinary tract infection or sexually transmitted infection, history of meconium-stained amniotic fluid, and history of intrapartum fever, respectively. In addition, 68 (23.5%) of the mothers had a history of diagnosed chorioamnionitis. Regarding the onset of labor, 226 (78.2%) of the mothers had spontaneous onset of labor, and 209 (72.3%), 52 (18%), and 28 (9.7%) of the mothers were delivered by spontaneous vaginal delivery, instrumental, and cesarean section, respectively, (Table 2).

### 3.3. Neonatal Characteristics

In considering neonatal characteristics, APGAR score, cry immediately at birth, resuscitation provision, kangaroo mother care practice, birth weight, and exclusive breastfeeding initiation within the first hour were observed. APGAR score was monitored for a total of 221 neonates. Among them, 41 (14.2%) had a score less than seven at the first minute and 39 (13.5%) of them had less than seven at the fifth minute. Among the dead neonates, 17 (68%) of them were neonates who have first minute APGAR score less than seven and 14 (56%) of the dead neonates were neonates who had fifth minute APGAR score less than seven. Nearly three fourths (74%) of the neonates were crying immediately at birth, 21 (7.3%) of the neonates were resuscitated at birth, and 229 (79.2%) of the neonates were kept under kangaroo mother care within the first hour of delivery.

In considering gestational age, 173 (59.9%) of the newborns were delivered at less than 37 weeks of gestation. The remaining 115 (39.8%) and one (0.3%) were delivered within 37-42 weeks and more than 42 weeks of gestation, respectively. In considering mortality, equal numbers of neonates (12, 48%) died among the neonates delivered at less than 37 weeks and 37-42 weeks of gestation. The minimum and maximum birth weight at admission was 785 grams and 5200 grams, respectively, with a mean of 2631.5 ± 877.2 grams. The majority of the newborn (56.1%) had a birth weight of more than 2500 grams. In addition, 60% of died had a birth weight less than 2500 grams. Regarding the initiation of exclusive breastfeeding, 177 (61.2%) of the neonates initiate within the first hour and three of dead the neonates were among the categories who did not initiate exclusive breastfeeding within the first hour (Table 3).

### 3.4. Incidence of Mortality among Neonates with Neonatal Sepsis

In this study, from the total (289) admitted culture-positive neonatal sepsis cases, 225 (77.9%) were admitted with early-onset neonatal sepsis (EONS) and 64 (22.1%) were with late-onset neonatal sepsis (LONS). The neonates were followed for a total of 1715 neonate days. Among them, a total of 25 neonates died. The incidence of mortality was 8.65 per 100 neonates admitted with neonatal sepsis, and the incidence rate of mortality among neonates admitted with neonatal sepsis was 14.57 per 1000 neonate days.

### 3.5. The Survival Status of Newborn Admitted with Neonatal Sepsis

**Figure 1:**
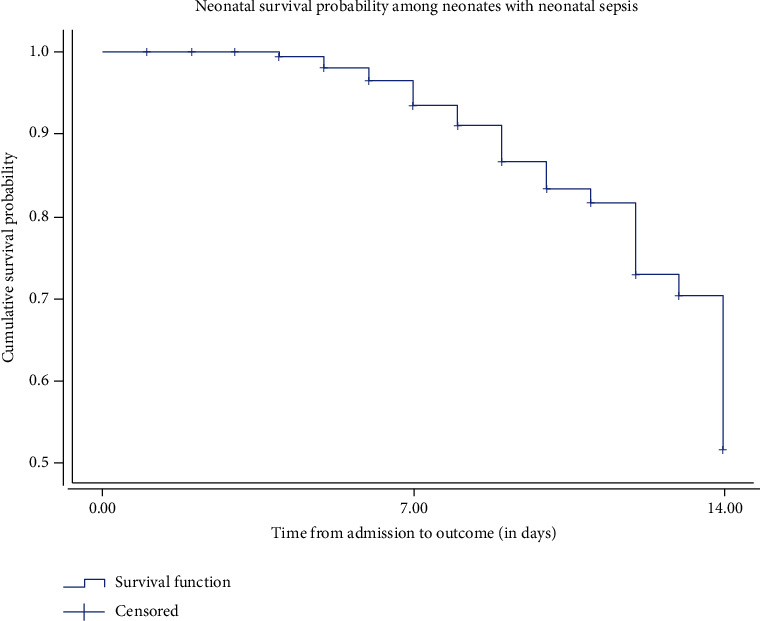
Neonatal survival status among neonates admitted with neonatal sepsis at public hospitals in Ethiopia.

Within the follow-up period, the first death was observed at the fourth day of the initiation of follow-up and the maximum follow-up period was 14 days. The cumulative proportion of surviving at the end of the fourth day of the initiation of follow up was 99.5% and 98.2% at the end of the fifth day. In addition, it was 96.6%, 93.5%, and 91.1% at the end of the sixth, seventh, and eighth day, respectively. As the follow-up period increased the survival probability decreases. The maximum observed follow-up period was 14 days, where the cumulative proportion of surviving was 51.7%. The overall mean survival time was 12.74 (95% CI: 12.234, 13.240). There was no observed death at the early follow-up period (first four days), and there was a gradual increment of hazard of death. At the late follow-up period, especially after the seven follow-up days, there was a rapid increment of neonatal mortality (Figure 1).

### 3.6. Log Rank Estimate of Mortality among Neonates with Neonatal Sepsis across Variables

The log-rank test estimate revealed that the survival pattern of mortality among neonates with neonatal sepsis was significantly varied among the covariates. The Kaplan-Meier survival curve together with log-rank test shows the effect of each variable on mortality of neonates with neonatal sepsis. Delivery attendant, history of foul-smelling liquor, history of UTI/STI, having history of intrapartum fever, and history of diagnosed chorioamnionitis were the variables, which had a high effect on neonatal mortality among neonates with neonatal sepsis (Table 4).

### 3.7. Comparison of Survivor Function between the Categories of Predictors of Mortality among Pediatrics with Neonatal Sepsis

In this study, since the maximum observation time was censored, the median survival time was not determined. Therefore, the mean survival time is the appropriate measures of central tendency. The overall mean survival time was 12.73 (95% CI: 12.23, 13.24) (Table 5).

### 3.8. Predictors of Mortality among Neonates with Neonatal Sepsis

Place of delivery, delivery attendant, number of per vaginal examinations, history of foul-smelling liquor, history of UTI/STI, having history of intrapartum fever, history of diagnosed chorioamnionitis, onset of labor, fifth minute APGAR score, and initiate EBF within one hour were variables which had *p* value ≤ 0.05 and analyzed using cox proportional hazard model. The multivariable analysis indicated that having a history of intrapartum fever, history of diagnosed chorioamnionitis, onset of labor, and initiate EBF within one hour were statistically significant in the final cox proportional hazard model.

Newborns who had delivered with mothers having a history of intrapartum fever were 14 times higher risk of death as compared with the counterparts (AHR: 14.5; 95% CI: 4.25, 49.5). The risk of mortality among newborns with neonatal sepsis was 5 times higher on those delivered with mothers having a history of diagnosed chorioamnionitis as compared with those with no history of no diagnosed chorioamnionitis (AHR: 5.7; 95% CI: 2.29, 13.98). Newborn with neonatal sepsis has 7 times higher hazard of mortality if labor is initiated by induction as compared with spontaneous initiation of labor (AHR: 7.0; 95% CI: 2.32, 21.08). The hazard of mortality among neonates with neonatal sepsis was 3 times higher if they did not initiate exclusive breastfeeding within one hour as compared with those who initiate within one hour (AHR: 3.4; 95% CI: 1.34, 12.63) (Table 6).

## 4. Discussion

This study determines the survival status and predictors of mortality among neonates admitted with neonatal sepsis at public hospitals in Ethiopia. Having a history of intrapartum fever, history of diagnosed chorioamnionitis, onset of labor, and initiate EBF within one hour were statistically significant predictors of mortality.

Consistent with the study conducted at the University of Gonder comprehensive specialized hospital, newborns who had delivered with mothers having a history of intrapartum fever were 14 times higher risk of death as compared with the counterparts (AHR: 14.5; 95% CI: 4.25, 49.5) [12]. This might be occurring due to the maternal preexisting infections [19]. Therefore, the disease-causing agent of maternal infection can ascend to the baby via circulation and during passage through the birth canal [20, 21].

In line with the study conducted at Tanzania, the risk of mortality among newborn with neonatal sepsis was 5 times higher on those delivered with mothers having a history of diagnosed chorioamnionitis as compared with those with no history of no diagnosed chorioamnionitis (AHR: 5.7; 95% CI: 2.29, 13.98) [5]. This might be due to the colonization of disease-causing agents to the birth canal and the vertical passage of the agent to the newborn during labor and delivery [22].

Newborn with neonatal sepsis has 7 times higher hazard of mortality if labor is initiated by induction as compared with spontaneous initiation of labor (AHR: 7.0; 95% CI: 2.32, 21.08). This might be due to prolonged gestation and premature membranes rupture. Prolonged gestation by itself had a risk of meconium aspiration, which leads to cause neonatal infection. For premature rupture of membranes (>37 weeks' gestation), it is recommended to offer induction of labor, or alternatively offering expectant management for a maximum of 24 hours and any longer increases the risk of ascending infection called as chorioamnionitis. Therefore, chorioamnionitis is considered as one cause of mortality among neonates with neonatal sepsis.

The hazard of mortality among neonates with neonatal sepsis was 3 times higher if they did not initiate exclusive breastfeeding within one hour as compared with those who did not initiate within one hour (AHR: 3.4; 95% CI: 1.34, 12.63). This finding is similar with the research conducted at developing countries [23]. This might be due to the reason breastfeeding controls the influence on the initial exposure of the newborn intestinal mucosa to microbes. Hence, limits disease-causing agents effect through the mucosa of the gut. In addition, the many defense factors of the mother's milk include large amounts of secretory immunoglobulin A (SIgA) antibodies produced by lymphocytes which have migrated from the mother's gut to the mammary glands. Therefore, the antibodies (secretory immune-globulins) were commonly directed against the maternal past and recent microflora of the gut [24].

In other sense, immediate initiation of exclusive breastfeeding acts as the initial point for a continuous maternal and newborn care that can have long-lasting effects on health and development. Therefore, early breastfeeding initiation provides adequate nutrition at an appropriate time, immunological value from first milk (colostrum), which prevents hypothermia and hypoglycemia [25].

In this study, place of delivery, delivery attendant, number of per vaginal examinations, history of urinary tract infections or sexually transmitted infections, and the fifth minute APGAR score were not statistically significant while they were significant in other studies conducted before. This indicates the need for meta-analysis. The limitation of this study was it does not determine the microbiological profile of the disease-causing agents.

## 5. Conclusion

The survival status of neonates among neonates admitted with neonatal sepsis was high at the early admission days and high cumulative proportion of death as the admission period increased. The risk of mortality was high among the neonates with early onset of neonatal sepsis as compared with late-onset of neonatal sepsis and having a history of intrapartum fever, history of diagnosed chorioamnionitis, onset of labor, and EBF initiation within one hour were the independent predictors of mortality among neonates admitted with neonatal sepsis. Therefore, mothers who had a history of fever and diagnosed chorioamnionitis need intensive supervision, and researchers need to conduct an evidence-based study for the initiation of antibiotics for those mothers. In addition, EBF should be in focus among the health professional, and mothers should be educated on the timing of initiation and its importance.

## Figures and Tables

**Table 1:** Sociodemographic characteristics of neonates with neonatal sepsis public hospitals in Ethiopia.

Variables	Category	Status at the end of the follow-up period	
		Died *n* (%)	Survived *n* (%)
Age of the newborn	<7 days	15 (60%)	210 (79.5)
	>7 days	10 (40%)	54 (20.5%)
Sex of the newborn	Male	16 (64.0%)	164 (62.1%)
	Female	9 (36.0%)	100 (37.9%)
Maternal age	<20	5 (20.0%)	33 (12.5%)
	20-34	9 (36.0%)	192 (72.7%)
	>34	11 (44.0%)	39 (14.8%)
Marital status	Never married	18 (72.0%)	9 (3.4%)
	Married	7 (28.0%)	255 (96.6%)
Religious status	Orthodox	11 (44.0%)	112 (42.4%)
	Muslim	5 (20.0%)	49 (18.6%)
	Protestant	7 (28.0%)	88 (33.3%)
	Others	2 (8.0%)	15 (5.7%)
Educational status of the mother	Unable to read and write	9 (36.0%)	30 (11.4%)
	Read and write	6 (24.0%)	60 (22.7%)
	Grade 1-8	5 (20.0%)	89 (33.7%)
	Grade 9-12	3 (12.0%)	56 (21.2%)
	College and above	2 (8.0%)	29 (11.0%)
Maternal occupational status	Housewife	8 (32.0%)	22 (8.3%)
	Self-employee	7 (28.0%)	63 (23.9%)
	Farmer	5 (20.0%)	90 (34.1%)
	Merchant	3 (12.0%)	62 (23.5%)
	Civil servant	2 (8.0%)	27 (10.2%)
Family size	<4	4 (16.0%)	127 (48.1%)
	4-6	6 (24.0%)	85 (32.2%)
	>6	15 (60.0%)	52 (19.7%)
Estimated monthly income of the family (ETB)	≤1399	10 (40.0%)	12 (4.5%)
	1400-1999	7 (28.0%)	65 (24.6%)
	2000-2599	5 (20.0%)	86 (32.6%)
	≥2600	3 (12.0%)	101 (38.3%)

**Table 2:** Obstetrics-related characteristics of mothers having neonates with neonatal sepsis at public hospitals in Ethiopia.

Variables	Category	Status at the end of the follow-up period	
		Died *n* (%)	Survived *n* (%)
Number of ANC visits	No	9 (36.0%)	22 (8.3%)
	One	6 (24.0%)	78 (29.5%)
	Two	5 (20.0%)	67 (25.4%)
	Three	3 (12.0%)	58 (22.0%)
	Four and above	2 (8.0%)	39 (14.8%)
Gravidity	<2	5 (20.0%)	87 (33.0%)
	2-4	9 (36.0%)	53 (20.1%)
	>4	11 (44.0%)	124 (47.0%)
Parity	<2	5 (20.0%)	87 (33.0%)
	2-4	9 (36.0%)	53 (20.1%)
	>4	11 (44.0%)	124 (47.0%)
Place of delivery	Health institution	9 (36.0)	212 (80.3)
	Home	16 (64.0%)	52 (19.7%)
Delivery attendant	Relatives	9 (36.0%)	22 (8.3%)
	TTBA	7 (28.0%)	30 (11.4%)
	HEW	5 (20.0%)	74 (28.0%)
	Health professionals	4 (16.0%)	138 (52.3%)
Number of per vaginal digital examination	<4	7 (28.0%)	164 (62.1%)
	>4	18 (72.0%)	100 (37.9%)
History of foul-smelling liquor	Yes	16 (64.0%)	29 (11.0%)
	No	9 (36.0%)	235 (89.0%)
History of pregnancy-induced hypertension	Yes	16 (64.0%)	55 (20.8%)
	No	9 (36.0%)	209 (79.2%)
History of bleeding during pregnancy	Yes	14 (56.0%)	61 (23.1%)
	No	11 (44.0%)	203 (76.9%)
History of UTI/STI	Yes	16 (64%)	38 (14.4%)
	No	9 (36.0%)	226 (85.6%)
History of meconium-stained amniotic fluid	Yes	25 (100%)	32 (12.1%)
	No	0 (0%)	232 (87.9%)
History of intrapartum fever	Yes	15 (60.0%)	19 (7.2%)
	No	10 (40.0%)	245 (92.8%)
History of diagnosed chorioamnionitis	Yes	18 (72.0%)	50 (18.9%)
	No	7 (28.0%)	214 (81.1%)
Time of rupture of membrane	<12hours	18 (72.0%)	84 (31.8%)
	>12 hours	7 (28.0%)	180 (68.2%)
Onset of labor	Spontaneous	13 (52.0%)	213 (80.7%)
	Induced	12 (48.0%)	51 (19.3%)
Mode of delivery	Spontaneous vaginal delivery	14 (56.0%)	195 (73.9%)
	Assisted instrumental delivery	9 (36.0%)	43 (16.3%)
	Cesarean section	2 (8.0%)	26 (9.8%)
Gestational age (weeks)	<37	12 (48.0%)	161 (61.0%)
	37-42	12 (48.0%)	103 (39.0%)
	>42	1 (4.0%)	0 (0%)

**Table 3:** Neonatal characteristics of newborn admitted with neonatal sepsis at public hospitals in Ethiopia.

Variables	Category	Status at the end of the follow-up period	
		Died *n* (%)	Survived *n* (%)
First minute APGAR score (*n* = 221)	<7	17 (68.0%)	24 (9.1%)
	>7	8 (32.0%)	172 (65.2%)
Fifth minute APGAR score (*n* = 221)	<7	14 (56.0%)	25 (9.5%)
	>7	7 (28.0%)	171 (64.8%)
Cry immediately at birth	Yes	17 (68.0%)	197 (74.6%)
	No	8 (32.0%)	67 (25.4%)
Resuscitated at birth	Yes	3 (12.0%)	18 (6.8%)
	No	22 (88.0%)	246 (93.2%)
Kept in KMC within one hour	Yes	19 (76.0%)	210 (79.5%)
	No	6 (24.0%)	54 (20.5%)
Birth weight (gram)	<2500	15 (60.0%)	112 (42.4%)
	>2500	10 (40.0%)	152 (57.6%)
EBF initiated within one hour	Yes	3 (12.0%)	174 (65.9%)
	No	22 (88.0%)	90 (34.1%)

**Table 4:** Log-rank estimate of variables on mortality of neonates with neonatal sepsis at public hospitals in Ethiopia.

Variables	Log-rank test estimate
Age of the newborn	*X* ^2^ = 0.007; *p* value = 0.93
Sex of the newborn	*X* ^2^ = 0.001; *p* value = 0.996
Maternal age	*X* ^2^ = 5.69; *p* value = 0.165
Marital status	*X* ^2^ = 4.24; *p* value = 0.241
Religious status	*X* ^2^ = 0.30; *p* value = 0.96
Educational status of the mother	*X* ^2^ = 7.25; *p* value = 0.12
Occupational status of the mother	*X* ^2^ = 8.21; *p* value = 0.08
Family size	*X* ^2^ = 1.993; *p* value = 0.81
Estimated monthly income	*X* ^2^ = 2.44; *p* value = 0.61
Number of ANC visits attended	*X* ^2^ = 6.65; *p* value = 0.15
Gravidity	*X* ^2^ = 1.70; *p* value = 0.42
Parity	*X* ^2^ = 3.33; *p* value = 0.189
Place of delivery	*X* ^2^ = 12.73; *p* value = 0.0001
Delivery attendant	*X* ^2^ = 23.39; *p* value = 0.0001
Number of PV	*X* ^2^ = 8.25; *p* value = 0.004
History of foul-smelling liquor	*X* ^2^ = 25.28; *p* value = 0.0001
History of PIH	*X* ^2^ = 1.27; *p* value = 0.821
History of bleeding during pregnancy	*X* ^2^ = 1.29; *p* value = 0.381
History of UTI/STI	*X* ^2^ = 34.27; *p* value = 0.0001
History of meconium-stained amniotic fluid	*X* ^2^ = 5.781; *p* value = 0.091
Having history of intrapartum fever	*X* ^2^ = 42.60; *p* value = 0.0001
History of diagnosed chorioamnionitis	*X* ^2^ = 27.44; *p* value = 0.0001
Onset of labor	*X* ^2^ = 5.37; *p* value = 0.02
Mode of delivery	*X* ^2^ = 4.37; *p* value = 0.112
Gestational age	*X* ^2^ = 2.40; *p* value = 0.30
First minute APGAR score	*X* ^2^ = 6.99; *p* value = 0.091
Cry immediately at birth	*X* ^2^ = 0.062; *p* value = 0.804
Fifth minute APGAR score	*X* ^2^ = 39.79; *p* value = 0.0001
Resuscitation at birth	*X* ^2^ = 0.03; *p* value = 0.86
Kept at KMC within 1 hour	*X* ^2^ = 0.001; *p* value = 0.979
Birth weight at admission	*X* ^2^ = 1.55; *p* value = 0.212
Initiate EBF within one hour	*X* ^2^ = 21.83; *p* value = 0.0001
Time of ROM occur	*X* ^2^ = 8.34; *p* value = 0.004

**Table 5:** The mean survival time and corresponding 95% CI among the covariates of predictors of mortality among neonates with neonatal sepsis.

Variables	Category	Mean survival time (95% CI)
Having history of intrapartum fever	Yes	10.29 (9.14, 11.44)
	No	13.46 (13.06, 13.87)
History of diagnosed chorioamnionitis	Yes	10.95 (9.78, 12.11)
	No	13.57 (13.19, 13.96)
Onset of labor	Spontaneous	12.99 (12.45, 13.54)
	Induced	12.09 (10.97, 13.22)
Initiate EBF within one hour	Yes	13.78 (13.41, 14.15)
	No	11.55 (10.67, 12.43)

**Table 6:** Predictors of mortality among neonates with neonatal sepsis at public hospitals in Ethiopia.

Variables	Category	Total	Neonatal death (%)	CHR (95% CI)	AHR (95% CI)
Place of delivery	Health institution	221	4.07%	1	1
	Home	68	23.5%	3.9 (1.69, 8.81)^∗^	1.2(0.22, 6.86)
Delivery attendant	Relatives	31	29.0%	10.7 (3.25, 34.92)^∗^	2.8 (0.51, 14.77)
	TTBA	18.9%	37	3.9 (1.13, 13.24)^∗^	1.2 (0.20, 6.59)
	HEW	6.3%	79	2.8 (0.74, 10.47)	1.4 (0.21, 9.98)
	Health professionals	2.8%	142	1	1
Number of PV	<4	4.1%	171	1	1
	>4	15.3%	118	3.3 (1.35, 7.81)^∗^	1.1 (0.36, 3.20)
History of foul-smelling liquor	Yes	35.6%	45	6.1 (2.66, 13.84)^∗^	1.1 (0.26, 4.86)
	No	3.7%	244	1	1
History of UTI/STI	Yes	29.6%	54	7.5 (3.30, 16.97)^∗^	0.7 (0.13, 3.69)
	No	3.8%	235	1	1
Having history of intrapartum fever	Yes	44.1%	34	9.3 (4.08, 21.04)^∗^	14.5 (4.25, 49.5)^∗∗^
	No	3.9%	255	1	1
History of diagnosed chorioamnionitis	Yes	26.5%	68	7.0 (2.92, 16.83)^∗^	5.7 (2.29, 13.98)^∗∗^
	No	3.2%	221	1	1
Onset of labor	Spontaneous	5.8%	226	1	1
	Induced	19.0%	63	2.4 (1.09, 5.26)^∗^	7.0 (2.32, 21.08)^∗∗^
Fifth minute APGAR score	<7	35.9%	39	8.7 (3.52, 21.68)^∗^	1.2 (0.39, 3.80)
	>7	3.9%	178	1	1
Initiate EBF within one hour	Yes	1.7%	177	1	1
	No	19.6%	112	9.8 (2.93, 33.00)^∗^	3.4 (1.34, 12.63)^∗∗^

^∗^Variable which had *p* value < 0.05 in bivariate analysis. ^∗∗^Variables which had *p* value < 0.05 in multivariable analysis.

## Data Availability

The data sets generated and/or analyzed are available with a reasonable request through the corresponding author.

## Abbreviations

ANC: Antenatal care
APGAR: Appearance, pulse, grimace, activity, and respiratory effort
EBF: Exclusive breastfeeding
SIgA: Secretory immunoglobulin A
STI: Sexually transmitted infection
UTI: Urinary tract infection
WHO: World Health Organization.
